# Risk of all–cause death and pancreatic events following GLP-1 RA initiation in people with obesity or type 2 diabetes: observations from a federated research network

**DOI:** 10.1186/s12933-025-02986-0

**Published:** 2025-11-19

**Authors:** Enrico Tartaglia, Tommaso Bucci, Michele Rossi, Andrea Galeazzo Rigutini, Amir Askarinejad, Uazman Alam, Katarzyna Nabrdalik, Giuseppe Boriani, Gregory Y. H. Lip

**Affiliations:** 1 Liverpool Centre for Cardiovascular Science at University of Liverpool, Liverpool John Moores University and Liverpool and Heart and Chest Hospital, Liverpool, UK; 2 Cardiology Division, Department of Biomedical, Metabolic and Neural Sciences, University of Modena and Reggio Emilia, Policlinico di Modena, Modena, Italy; 3 Department of Life, Health & Environmental Sciences, University of L’Aquila, L’Aquila, Italy; 4 Internal Medicine and Nephrology Division, ASL1 Avezzano-Sulmona-L’Aquila, San Salvatore Hospital, L’Aquila, Italy; 5 Internal, Vascular and Emergency Medicine – Stroke Unit, University of Perugia, Perugia, Italy; 6 Department of Cardiovascular and Metabolic Medicine, University of Liverpool, Liverpool, UK; 7 Department of Medicine, University Hospital Aintree, Liverpool University NHS Foundation Trust, Liverpool, UK; 8 Centre for Biomechanics and Rehabilitation Technologies, Staffordshire University, Stoke-on-Trent,, UK; 9 Department of Internal Medicine, Diabetology and Nephrology, Faculty of Medical Sciences in Zabrze, Medical University of Silesia, Katowice, Poland; 10 University of Modena and Reggio Emilia, Modena, Italy; 11 Department of Clinical Medicine, Aalborg University, Aalborg, Denmark; 12 Department of Cardiology, Lipidology and Internal Medicine, Medical University of Bialystok, Bialystok, Poland

**Keywords:** GLP-1 receptor agonists, Type 2 diabetes, Obesity, Pancreatic outcomes, Death, Propensity score matching

## Abstract

**Background:**

Limited data are available on the risk of pancreatic adverse events among people with obesity or type 2 diabetes mellitus (T2DM) initiating glucagon-like peptide-1 receptor agonist (GLP-1 RA).

**Methods:**

Retrospective study utilizing data from a federated research network (TriNetX). Adult people (≥ 18 years) with a diagnosis of obesity (body mass index ≥ 30 kg/m^2^) or T2DM (ICD-10-CM: E11) between 2018 and 2024 were subdivided in two mutually exclusive cohorts: (1) GLP-1 RA Users; and (2) Non–GLP-1 RA Users. Primary outcomes were 1-year risk of all-cause death and a composite outcome (acute pancreatitis, chronic pancreatitis). Secondary outcomes included the individual components of the composite outcome and pancreatic cancer. Cox regression analyses were employed to calculate hazard ratios (HRs) and 95% confidence intervals (CIs) before and after 1:1 propensity score matching (PSM). Sensitivity analyses stratified follow-up into early (first 6 months) and late (last 6 months) phases. Subgroup analyses were performed based on age (≥ 65 or < 65 years), sex, and the history of smoking and alcohol use, hypertriglyceridemia, cholelithiasis, heart failure, and chronic kidney disease.

**Results:**

We identified 1,562,626 people who initiated treatment with GLP-1 RA (mean age 55.3 ± 14.0 years; 59.2% female) and 18,652,572 those who did not (mean age 50.6 ± 18.7 years; 54.5% female). Before PSM, GLP-1 RA Users were older, more frequently female, and exhibited a higher burden of endocrine, metabolic and gastrointestinal disorders. After PSM, GLP-1 RA use was associated with a substantially lower risk of all-cause death (HR 0.554, 95% CI 0.542–0.566), and small increased risk of the composite outcome (HR 1.062, 95% CI 1.023–1.102) and acute pancreatitis (HR 1.058, 95% CI 1.015–1.103), with no differences in chronic pancreatitis or pancreatic cancer. The excess risk of acute pancreatitis was more pronounced during the early phase of follow-up (first 6 months). Subgroup analyses showed a higher reduction in death and composite outcome among people aged < 65 years. Additional significant interactions were observed for all-cause death in females and in people with a history of smoking, alcohol use, heart failure, chronic kidney disease, or cholelithiasis.

**Conclusion:**

GLP-1 RA use was associated with substantially reduced all-cause death but a small increased risk of acute pancreatitis, particularly during early treatment. The survival benefit was more pronounced in younger people and those with cardiometabolic comorbidities, highlighting the need for a careful risk–benefit evaluation when prescribing GLP-1 RAs in high-risk individuals.

**Supplementary Information:**

The online version contains supplementary material available at 10.1186/s12933-025-02986-0.

## Research Insights

**What is currently known about this topic? **GLP-1 RAs improve glycemic control and weight. GLP-1 RAs reduce cardiovascular risk in T2DM/obesity. Concerns remain about pancreatic safety

**What is the key research question?** Do GLP-1 RAs increase pancreatic risk while lowering death?

**What is new?** GLP-1 RAs lower all-cause death in real-world data. Early higher risk of acute pancreatitis with GLP-1 RAs

**How might this study influence clinical practice?** Supports careful risk–benefit assessment before prescribing

## Introduction

Glucagon-like peptide-1 receptor agonists (GLP-1 RAs) are a class of incretin-based therapies that mimic the action of endogenous GLP-1, a gut-derived hormone secreted in response to nutrient ingestion [1]. By binding to GLP-1 receptors on pancreatic β-cells, these agents enhance glucose-dependent insulin secretion while suppressing glucagon release from α-cells, thereby improving glycaemic control in a physiologically regulated manner [1]. Beyond their pancreatic effects, GLP-1 RAs act on receptors in the hypothalamus and gastrointestinal tract to delay gastric emptying, reduce appetite, and increase satiety [1].

Owing to this dual mechanism of action, GLP-1 RAs have become the cornerstone of management of type 2 diabetes mellitus (T2DM), particularly in people with inadequate glycaemic control despite standard therapies [2]. More recently, their indications have expanded to individuals with obesity—even in the absence of diabetes—based on compelling evidence of clinically meaningful weight loss and metabolic improvement [3, 4].

Across both T2DM and obesity, GLP-1 RAs have consistently demonstrated broad cardiometabolic benefits [1]. In addition to improvement of blood glucose control and body weight, these agents have been shown to lower blood pressure, improve lipid profiles, and attenuate systemic inflammation [5, 6]. Large cardiovascular outcome trials with the use of liraglutide, semaglutide and dulaglutide [7] performed in people with T2DM as well as with semaglutide performed in people with obesity [8] have further confirmed their ability to reduce major adverse cardiovascular events—including myocardial infarction, stroke, and cardiovascular death—solidifying their role as disease-modifying therapies at the intersection of metabolic and cardiovascular care [9, 10].

Nonetheless, as GLP-1 RAs are increasingly prescribed across a wide range of populations, concerns have arisen regarding their long-term safety—particularly in relation to pancreatic health. Preclinical studies and pharmacovigilance reports have suggested a possible association with pancreatic diseases, such as acute and chronic pancreatitis and pancreatic cancer [11, 12]. While biologically plausible, these risks remain unconfirmed, as clinical trials were not powered to assess pancreatic safety, and real-world evidence is limited.

To address this knowledge gap, we conducted a large retrospective cohort study using data from a global federated research network to evaluate the risk of all-cause death and pancreatic adverse events in people with obesity or T2DM initiating GLP-1 RA therapy.

## Methods

### Study design

This retrospective observational study was conducted using the TriNetX Research Network (TriNetX, Cambridge, MA, USA), a global federated health research network that provides access to anonymized electronic medical records from over 130 healthcare organizations, including academic medical centres, community hospitals, and outpatient clinics. The network includes more than 300 million individuals, primarily in the United States (U.S.).

Available data include demographics, diagnoses coded using the International Classification of Diseases, Ninth and Tenth Revisions, Clinical Modification (ICD-9-CM and ICD-10-CM) codes, and medications coded with Veteran Affairs (VA) codes or RxNorm classification systems, depending on the contributing institution.

Further information about the network is available online (https://trinetx.com/company-overview/).

TriNetX operates in compliance with the Health Insurance Portability and Accountability Act (HIPAA) and US federal law. All patient data are de-identified in accordance with HIPAA’s Privacy Rule, and no patient-level identifiers are accessible. Access to the data is governed through formal data-sharing agreements with participating institutions. As a federated network using only de-identified data, studies conducted within TriNetX do not require approval from institutional review boards or informed consent. Additional methodological details are provided in the Supplementary Material.

### Cohort definition

The searches on the TriNetX online research platform were performed on October 10, 2025, using the U.S. Collaborative Network. At the time of data extraction, 66 healthcare organizations across the U.S. had contributed eligible patient data.

Eligibile criteria were as follows: aged 18 years or older, diagnosis of obesity, defined as a body mass index (BMI) ≥ 30 kg/m^2^ or T2DM (ICD-10-CM: E11), between January 1, 2018, and January 1, 2024.

Two mutually exclusive cohorts were constructed. (1) The first cohort (GLP-1 RA Users) included people who had received a prescription for a GLP-1 RA (ATC code: A10BJ, including liraglutide, semaglutide, dulaglutide, exenatide, lixisenatide, and albiglutide). (2) The second cohort (Non-GLP-1 RA Users) included people who met the same clinical criteria but had no record of GLP-1 RA use.

To reduce confounding from underlying pancreatic conditions, exclusion criteria included people with any prior diagnosis of acute pancreatitis (ICD-10-CM: K85), chronic pancreatitis (K86.0 or K86.1), or pancreatic cancer (C25) recorded before the index event.

The index event was defined as the first date on which the cohort-specific eligibility criteria were met.

A detailed list of inclusion and exclusion criteria, coding, and temporal logic used in the cohort construction is provided in Supplementary Table S1.

### Outcomes

The primary outcomes were the 1-year risk of all-cause death and a composite outcome comprising acute pancreatitis and chronic pancreatitis.

Secondary outcomes included each individual component of the primary composite outcome (acute pancreatitis; chronic pancreatitis) and pancreatic cancer.

To specifically assess potential drug-related effects of GLP-1 RA, follow-up for all outcomes began on day 30 after the index event. This was chosen to avoid misattributing outcomes to pre-existing conditions or events unrelated to the pharmacological effect. The observation window extended through day 395 to reflect 1-year follow-up.

Events were identified using ICD-10-CM diagnosis codes and recorded vital status (see Supplementary Table S2 for the complete code list).

### Statistical analysis

Baseline characteristics of GLP-1 RA Users and Non–GLP-1 RA Users were compared and balanced using logistic regression and 1:1 propensity score matching (PSM). Matching was performed using the greedy nearest neighbour method, employing a calliper of 0.1.

The following covariates were included in the PSM model: age, sex, ethnicity, diagnosis of endocrine, nutritional and metabolic disorders (ICD-10-CM: E00–E89, including disorders of thyroid gland, other endocrine glands, glucose regulation and pancreatic internal secretion, overweight and obesity, nutritional deficiencies, malnutrition, postprocedural and intraoperative endocrine complications), and diagnosis of disorders of the gastrointestinal system (ICD-10-CM: K00–K95, including disorders of the oral cavity, esophagus, stomach, intestines, liver, pancreas, gallbladder, hernia, and other gastrointestinal disorders). These variables were selected based on their relevance to GLP-1 RA prescription practices and their potential influence on pancreatic outcomes or death. The balance of demographics and clinical variables between the groups was assessed using Absolute Standardized Mean Differences (ASD), with an ASD of less than 0.1 indicating adequate balance.

Cox proportional hazards models, before and after PSM, were used to calculate hazard ratios (HRs) and 95% confidence intervals (95% CIs) for the risk of adverse events in GLP-1 RA Users compared to Non-GLP-1 RA Users.

Aalen-Johansen curves were used to represent the daily cumulative incidence of secondary outcomes, accounting for all-cause death as a competing risk, in GLP-1 RA Users compared to Non-GLP-1 RA Users.

The proportional hazards assumption was tested for each outcome using Schoenfeld residuals and corresponding Chi-square (χ^2^) tests. Further details on the performance and interpretation of this test can be found under the “Supplementary Methods” section of the Supplementary Material.

When the proportional hazards assumption was not met in the primary analysis, we performed additional time-stratified analyses by subdividing the 1-year follow-up period into two phases: an early phase (the first 6 months, from day 30 to day 210) and a late phase (the last 6 months, from day 211 to day 395). We then reassessed the risk using Cox regression and re-tested the proportional hazards assumption for each phase. This approach was selected to yield clinically interpretable estimates of early versus late risk, particularly when comparing exposure to pharmacological therapy with non-exposure. Acknowledging that two fixed intervals represent a simplification, the analysis was complemented with supplementary time-stratified assessments using alternative time windows—day 30–120 (0–3 months) and day 30–210 (0–9 months)—in addition to the pre-specified 0–6 and 6–12-month intervals, thereby enhancing temporal resolution without sacrificing interpretability; these additional analyses are provided in the Supplementary Material.

To reinforce the primary analyses, two complementary analyses were conducted using the same eligibility window, exclusions, index definition, outcomes, and follow-up as the main analysis.

First, the comparison between GLP-1 RA Users and Non–GLP-1 RA Users was repeated using an alternative propensity-score model based on markers of diabetes severity (baseline HbA1c and ICD-10-CM codes for type 2 diabetes with kidney [E11.2], neurological [E11.4], circulatory [E11.5] complications, and ketoacidosis [E11.1]), reflecting platform constraints that allow only a limited number of covariates to be modelled simultaneously.

Second, an active-comparator, new-user analysis compared GLP-1 RA Users with SGLT2 inhibitor Users (ATC A10BK) defined analogously (i.e., adults meeting the same eligibility criteria with a prescription for an SGLT2 inhibitor; identical exclusions; index as the first date on which cohort-specific eligibility criteria were met). Propensity-score matching used 1:1 greedy nearest neighbour with a 0.1 caliper; the first model employed the severity covariates listed above, whereas the active-comparator analysis used the same covariate set as in the main analysis. For both analyses, Cox proportional hazards models after PSM were used to estimate HRs and 95% CIs for the risk of adverse events.

Pre-specified subgroup analyses were performed as exploratory analyses to explore potential effect modification on the primary outcomes. Subgroups were defined according to: (1) patient demographic characteristics, including age (people aged ≥ 65 years vs. < 65 years) and sex (female vs. male); and (2) baseline clinical conditions, including the presence or absence of history of smoking, alcohol use, hypertriglyceridemia, cholelithiasis, heart failure, and chronic kidney disease.

For each subgroup comparison, separate propensity score matching, and survival analyses were performed using the same matching criteria and statistical methods described for the primary analysis.

The construction of each cohort and the corresponding ICD-10-CM codes for inclusion and exclusion are reported in Supplementary Table S1.

All statistical analyses were performed with the TriNetX analytics platform, which integrates R (v3.2–3) and Python 3.7 libraries (scikit-learn for logistic models; survival for Cox analysis). TriNetX does not replace missing values. All p-values were two-tailed, with statistical significance set at *p* < 0.05.

## Results

The final cohort included 1,562,626 people who received GLP-1 RA (mean age 55.3 ± 14.0 years; 59.2% female) and 18,652,572 people who did not receive GLP-1 RA (mean age 50.6 ± 18.7 years; 54.5% female) (Table 1).

**Table 1:** Baseline characteristics of people with obesity or type 2 diabetes mellitus who received a GLP-1 receptor agonist prescription (GLP-1 RA Users) compared to those who did not (Non–GLP-1 RA Users)

	Pre PSM			After PSM		
	GLP-1 RA Users (n = 1,562,626)	Non–GLP-1 RA Users (n = 18,652,572)	ASD	GLP-1 RA Users (n = 1,562,626)	Non–GLP-1 RA Users (n = 1,562,626)	ASD
Age at Index, mean ± SD	55.3 ± 14.0	50.6 ± 18.7	0.285	55.3 ± 14.0	55.3 ± 14.0	< 0.001
Female, n (%)	925,416 (59.2)	10,164,018 (54.5)	0.096	925,416 (59.2)	925,416 (59.2)	< 0.001
Not Hispanic or Latino, n (%)	1,072,852 (68.7)	12,096,591 (64.9)	0.081	1,072,852 (68.7)	1,072,852 (68.7)	< 0.001
Endocrine and metabolic disorders, n (%)	1,324,815 (84.8)	5,568,141 (29.9)	1.335	1,324,815 (84.8)	1,324,815 (84.8)	< 0.001
Gastrointestinal disorders, n (%)	799,571 (51.2)	3,890,179 (20.9)	0.666	799,571 (51.2)	799,571 (51.2)	< 0.001
Type 2 diabetes mellitus, n (%)	915,446 (60.0%)	1,433,597 (7.9%)	1.331	915,446 (60.0%)	915,446 (60.0%)	< 0.001
Obesity, n (%)	503,464 (33.2%)	1,240,102 (6.8%)	0.700	503,464 (33.2%)	503,464 (33.2%)	< 0.001
Hyperlipidemia, n (%)	625,951 (41.3%)	2,098,421 (11.5%)	0.718	625,951 (41.3%)	625,951 (41.3%)	< 0.001
Hyperglyceridemia, n (%)	61,908 (4.1%)	137,248 (1.0%)	0.218	61,908 (4.1%)	61,908 (4.1%)	< 0.001
Hyperthyroidism, n (%)	22,644 (2.0%)	101,136 (1.0%)	0.093	22,644 (2.0%)	22,644 (2.0%)	< 0.001
Hypothyroidism, n (%)	217,998 (14.4%)	870,261 (5.0%)	0.311	217,998 (14.4%)	217,998 (14.4%)	< 0.001
Disorders of upper GI tract, n (%)	462,072 (31.0%)	1,837,141 (10.0%)	0.364	462,072 (31.0%)	462,072 (31.0%)	< 0.001
Liver disorders, n (%)	159,006 (13.0%)	477,342 (3.0%)	0.375	159,006 (13.0%)	159,006 (13.0%)	< 0.001
Cholelithiasis, n (%)	53,490 (4.0%)	223,649 (1.2%)	0.152	53,490 (4.0%)	53,490 (4.0%)	< 0.001
Cholecystitis, n (%)	14,480 (1.0%)	61,485 (0.3%)	0.077	14,480 (1.0%)	14,480 (1.0%)	< 0.001
Alcohol use, n (%)	15,626 (1.0%)	18,652 (0.1%)	0.122	15,626 (1.0%)	15,626 (1.0%)	< 0.001
Smoking, n (%)	15,626 (1.0%)	18,652 (0.1%)	0.122	15,626 (1.0%)	15,626 (1.0%)	< 0.001
Oral hypoglycaemic agents, n (%)	827,469 (55.0%)	1,256,927 (7.0%)	1.208	827,469 (55.0%)	827,469 (55.0%)	< 0.001
Metformin, n (%)	709,679 (46.8%)	1,038,923 (5.7%)	1.057	709,679 (46.8%)	709,679 (46.8%)	< 0.001
Insulin, n (%)	494,764 (32.7%)	1,019,517 (5.6%)	0.733	494,764 (32.7%)	494,764 (32.7%)	< 0.001
Glipizide, n (%)	156,412 (11.0%)	215,244 (1.1%)	0.418	156,412 (11.0%)	156,412 (11.0%)	< 0.001
Empagliflozin, n (%)	148,584 (10.4%)	146,617 (0.8%)	0.465	148,584 (10.4%)	148,584 (10.4%)	< 0.001
Dapagliflozin, n (%)	63,443 (4.4%)	126,913 (0.7%)	0.290	63,443 (4.4%)	63,443 (4.4%)	< 0.001
Canagliflozin, n (%)	41,597 (2.8%)	57,960 (0.1%)	0.226	41,597 (2.8%)	41,597 (2.8%)	< 0.001
Linagliptin, n (%)	35,828 (2.4%)	40,755 (0.2%)	0.197	35,828 (2.4%)	35,828 (2.4%)	< 0.001
Sitagliptin, n (%)	132,936 (9.3%)	154,127 (0.8%)	0.393	132,936 (9.3%)	132,936 (9.3%)	< 0.001

PSM indicates Propensity Score Matching; ASD indicates Absolute Standardized mean Difference

Before PSM, GLP-1 RA Users were older, more frequently female, and exhibited a substantially higher prevalence of endocrine, nutritional and metabolic disorders, as well as gastrointestinal system disorders, compared to Non–GLP-1 RA Users (Table 1).

The number of primary and secondary outcomes, along with corresponding HRs for the comparison before and after PSM, are reported in Table 2.

**Table 2:** Risks of primary and secondary outcomes in people with obesity or type 2 diabetes mellitus who received a GLP-1 receptor agonist prescription (GLP-1 RA Users) compared to those who did not (Non–GLP-1 RA Users)

	Pre PSM			After PSM		
	GLP-1 RA Users (1,562,626)	Non–GLP-1 RA Users (18,652,572)	HR (95% CI)	GLP-1 RA Users (1,562,626)	Non–GLP-1 RA Users (1,562,626)	HR (95% CI)
All-cause death, n (%)	13,410 (0.9)	250,102 (1.3)	0.578 (0.568, 0.588)	13,410 (0.9)	25,127 (1.6)	0.554 (0.542, 0.566)
Composite outcome, n (%)	5514 (0.35)	39,020 (0.21)	1.524 (1.481, 1.567)	5514 (0.35)	5363 (0.34)	1.062 (1.023, 1.102)
Acute pancreatitis, n (%)	4501 (0.29)	31,953 (0.17)	1.519 (1.473, 1.568)	4501 (0.29)	4397 (0.28)	1.058 (1.015, 1.103)
Chronic pancreatitis, n (%)	1483 (0.1)	10,906 (0.06)	1.464 (1.387, 1.546)	1483 (0.1)	1573 (0.1)	0.973 (0.906, 1.045)
Pancreatic cancer, n (%)	1213 (0.08)	10,781 (0.06)	1.210 (1.140, 1.284)	1213 (0.08)	1207 (0.08)	1.031 (0.952, 1.116)

PSM indicates Propensity Score Matching; HR indicates Hazard Ratio; CI indicates Confidence Interval; n (%) indicates the number of events, with the percentage in parentheses calculated over the total size of the corresponding cohort

In the unmatched cohort, GLP-1 Users showed a lower risk of all-cause death (HR 0.578, 95% CI 0.568–0.588) compared to Non–GLP-1 RA Users, but a higher risk of the composite outcome (HR 1.524, 95% CI 1.481–1.567) (Table 2). Among secondary outcomes, GLP-1 RA Users exhibited a higher risk of acute pancreatitis (HR 1.519, 95% CI 1.473–1.568), chronic pancreatitis (HR 1.464, 95% CI 1.387–1.546), and pancreatic cancer (HR 1.210, 95% CI 1.140–1.284), compared to Non–GLP-1 RA Users (Table 2).

**Fig. 1:**
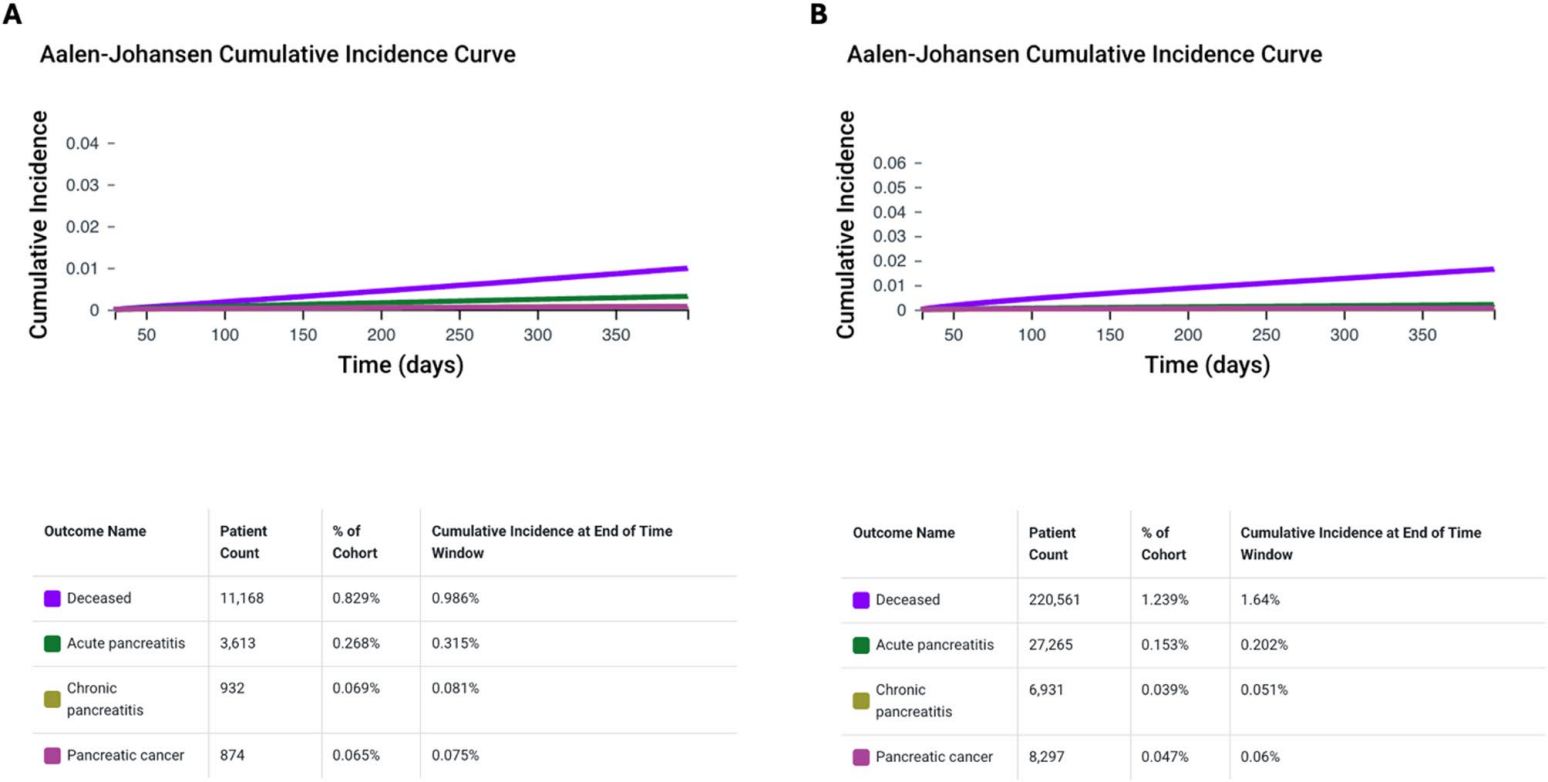
Aalen-Johansen curves before propensity score matching for the cumulative incidence of primary and secondary outcomes in people with obesity or type 2 diabetes mellitus who received a GLP-1 receptor agonist prescription (GLP-1 RA Users, Panel A) and those who did not (Non–GLP-1 RA Users, Panel B)

Aalen-Johansen curves before PSM in GLP-1 RA Users and Non–GLP-1 RA Users are shown in Fig. 1. The 1-year cumulative incidences for primary and secondary outcomes were as follows: 0.99% vs. 1.64% for all-cause death; 0.32% vs. 0.20% for acute pancreatitis; 0.08% vs. 0.05% for chronic pancreatitis; and 0.08% vs. 0.06% for pancreatic cancer in GLP-1 RA Users and Non–GLP-1 RA Users, respectively (Fig. 1, Panels A and B).

After PSM, baseline characteristics—including demographics, endocrine, metabolic and gastrointestinal system disorders—were well balanced across groups, with all ASD below 0.1 (Table 1).

In the matched cohort, GLP-1 RA Users continued to show a lower risk of all-cause death (HR 0.554, 95% CI 0.542–0.566) and a higher risk of the composite outcome (HR 1.062, 95% CI 1.023–1.102) compared to Non–GLP-1 RA Users (Table 2).

Among secondary outcomes, GLP-1 RA Users showed a higher risk of acute pancreatitis (HR 1.058, 95% CI 1.015–1.103), while no statistically significant differences were observed for chronic pancreatitis (HR 0.973, 95% CI 0.906–1.045) or pancreatic cancer (HR 1.031, 95% CI 0.952–1.116) (Table 2).

**Fig. 2:**
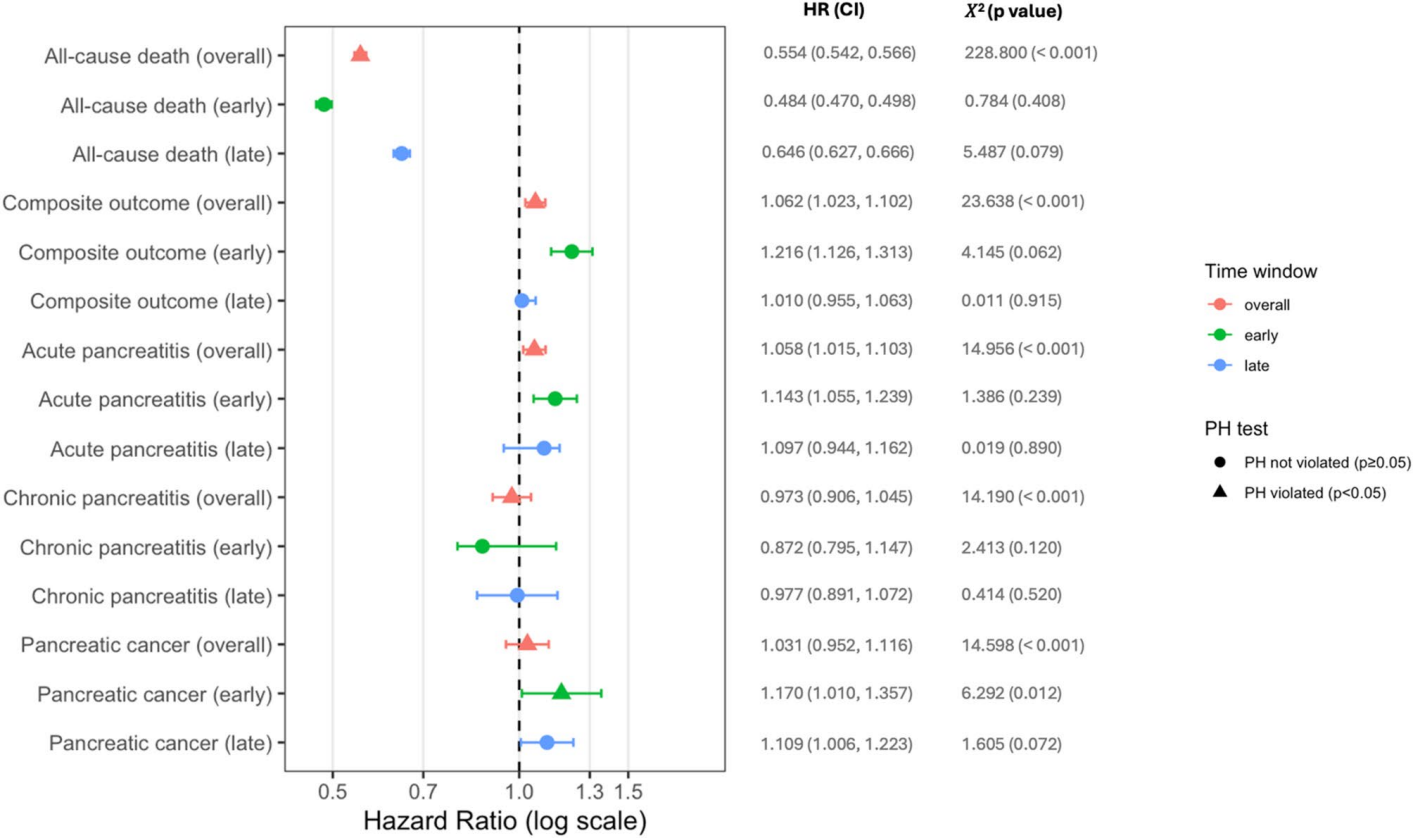
Risk of primary and secondary outcomes in people with obesity or type 2 diabetes mellitus who received a GLP-1 receptor agonist prescription (GLP-1 RA Users) compared to those who did not (Non–GLP-1 RA Users) across overall, early (the first 6 months) and late (the last 6 months) follow-up phases. HR indicates Hazard Ratios; CI indicates confidence interval. A high χ^2^ value suggests a greater deviation from the expected values, indicating a potential violation of the proportional hazard assumption. Conversely, a small χ^2^ value indicates that the observed residuals closely align with the expected values

When testing the proportional hazards assumption for the 1-year risk of all primary and secondary outcomes in GLP-1 RA Users compared to Non–GLP-1 RA Users after PSM, deviations from proportionality were observed for all outcomes (Fig. 2; Supplementary Table 3).

Specifically, the proportional hazards assumption was violated for all-cause death (χ^2^ = 228.800, *p* < 0.001), the composite outcome (χ^2^ = 23.638, *p* < 0.001), acute pancreatitis (χ^2^ = 14.956, *p* < 0.001), chronic pancreatitis (χ^2^ = 14.190, *p* < 0.001), and pancreatic cancer (χ^2^ = 14.598, *p* < 0.001) (Fig. 2; Supplementary Table 3). Hence, when the 1-year follow-up period was subdivided into two distinct phases, we observed temporal differences in the associations between GLP-1 RA use and study outcomes.

During the early phase (first 6 months, from day 30 to day 210), GLP-1 RA Users demonstrated a lower risk of all-cause death (HR 0.484, 95% CI 0.470–0.498) but a higher risk of the composite outcome (HR 1.216, 95% CI 1.126–1.313), acute pancreatitis (HR 1.143, 95% CI 1.055–1.239), and pancreatic cancer (HR 1.170, 95% CI 1.010–1.357), compared to Non–GLP-1 RA Users. No significant difference was observed for chronic pancreatitis (HR 0.872, 95% CI 0.795–1.147) (Fig. 2, Supplementary Table 3).

In the late phase (last 6 months, from day 211 to day 395), GLP-1 RA Users maintained a reduced risk of all-cause death (HR 0.646, 95% CI 0.627–0.666) and a higher risk of pancreatic cancer (HR 1.109, 95% CI 1.006–1.223). However, no significant associations were detected for the composite outcome (HR 1.010, 95% CI 0.955–1.063), acute pancreatitis (HR 1.097, 95% CI 0.944–1.162) or chronic pancreatitis (HR 0.977, 95% CI 0.891–1.072) (Fig. 2, Supplementary Table 3).

The proportional hazards assumption was met for all outcomes in both phases, with the exception of pancreatic cancer in the early phase (χ^2^ = 6.292, *p* = 0.012) (Fig. 2, Supplementary Table 3).

As previously described in the Methods, the results of the additional time-stratified analyses using alternative time windows (day 30–120 and day 30–270), including HRs with 95% CIs for each outcome after PSM, are presented in Supplementary Table 4.

Regarding the complementary analyses, the results were directionally consistent with the primary results and are summarized below.

*Diabetes-severity model (GLP-1 RA Users vs Non–GLP-1 RA Users)*: GLP-1 RA Users had a lower risk of all-cause death (HR 0.363, 95% CI 0.355–0.370) and a higher risk of the composite outcome (HR 1.047, 95% CI 1.007–1.088) (Supplementary Table 5). Among secondary outcomes, the risk of acute pancreatitis was higher (HR 1.068, 95% CI 1.023–1.114), whereas chronic pancreatitis (HR 0.933, 95% CI 0.867–1.005) and pancreatic cancer (HR 0.953, 95% CI 0.880–1.033) did not differ significantly from Non–GLP-1 RA Users (Supplementary Table 5).

*Active-comparator analysis (GLP-1 RA Users vs SGLT2 inhibitor Users)*: GLP-1 RA Users showed a lower risk of all-cause death (HR 0.694, 95% CI 0.679–0.710) and a higher risk of the composite outcome (HR 1.086, 95% CI 1.049–1.124) (Supplementary Table 5). For secondary outcomes, risks were higher for acute pancreatitis (HR 1.054, 95% CI 1.014–1.096), with no significant difference in chronic pancreatitis (HR 1.166, 95% CI 0.970–1.240) and pancreatic cancer (HR 0.981, 95% CI 0.912–1.055) compared to SGLT2 inhibitor Users (Supplementary Table 5).

### Subgroup analyses

**Fig. 3:**
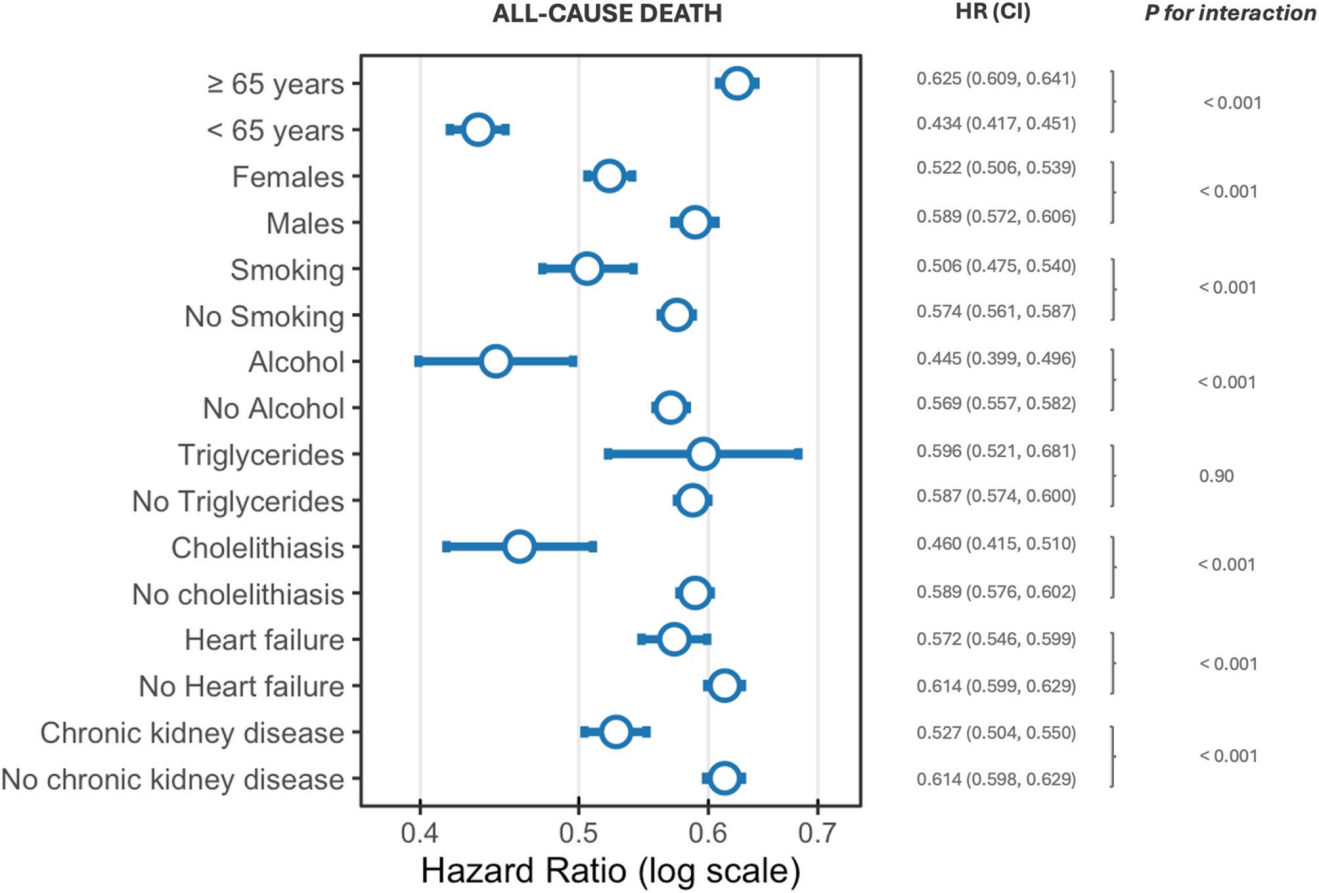
Risks of all-cause death in people with obesity or type 2 diabetes mellitus who received a GLP-1 receptor agonist prescription (GLP-1 RA Users) compared to those who did not (Non–GLP-1 RA Users) based on age (≥ 65 years vs. < 65 years), sex (female vs male) and presence or absence of history of tobacco use, alcohol use, hypertriglyceridemia, cholelithiasis, heart failure, and chronic kidney disease. Hazard Ratios (HRs) with 95% confidence intervals (CIs) shown in parentheses are shown for each subgroup. *P* values for interaction between subgroups are reported beneath each outcome label

**Fig. 4:**
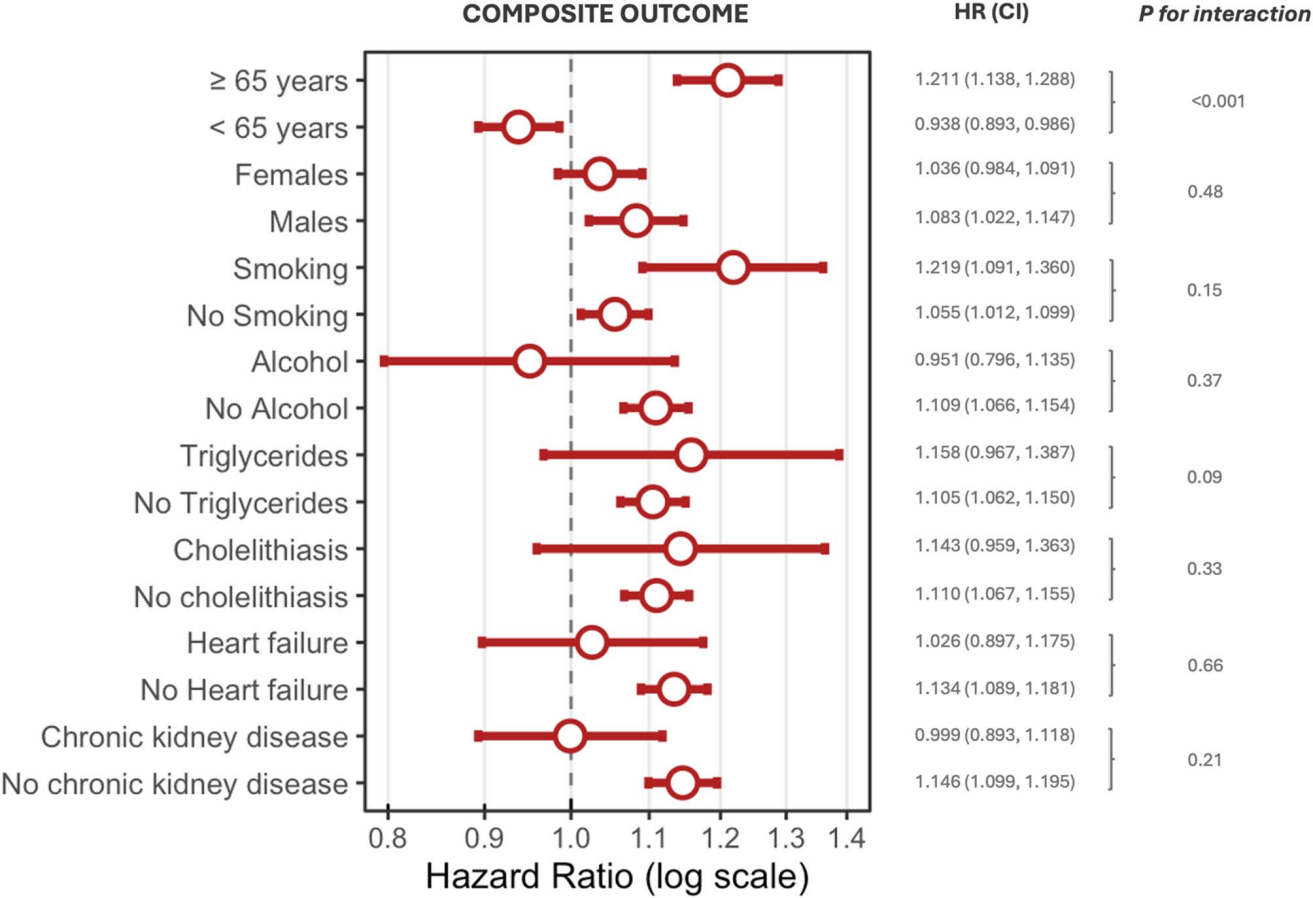
Risks of composite outcome in people with obesity or type 2 diabetes mellitus who received a GLP-1 receptor agonist prescription (GLP-1 RA Users) compared to those who did not (Non–GLP-1 RA Users) based on age (≥ 65 years vs. < 65 years), sex (female vs male) and presence or absence of history of tobacco use, alcohol use, hypertriglyceridemia, cholelithiasis, heart failure, and chronic kidney disease. Hazard Ratios (HRs) with 95% confidence intervals (CIs) shown in parentheses are shown for each subgroup. *P* values for interaction between subgroups are reported beneath each outcome label

A significant interaction by age (≥ 65 years vs. < 65 years) was observed for both all-cause death (HR 0.625, 95% CI 0.609–0.641 vs. HR 0.434, 95% CI 0.417–0.451; *p* for int < 0.001) and the composite outcome (HR 1.211, 95% CI 1.138–1.288 vs. HR 0.938, 95% CI 0.893–0.986; *p* for int < 0.001), indicating a less pronounced reduction in all-cause death but a greater increase in the composite risk among GLP-1 RA Users aged ≥ 65 years compared with those aged < 65 years (Figs. 3, 4, Supplementary Table 6).

For all-cause death, additional significant interactions were identified across subgroups defined by female sex, positive history of smoking and alcohol use, presence of heart failure, chronic kidney disease, and history of cholelithiasis (all *p* for interaction < 0.001) (Fig. 3; Supplementary Table 6). In each case, GLP-1 RA use was associated with a more substantial reduction in death among people with the respective risk factor.

In contrast, no significant interactions were found for the composite outcome across these same subgroups (*p* for int > 0.05) (Fig. 4; Supplementary Table 6).

## Discussion

In our study, four principal findings emerged: **(1)** People receiving GLP-1 RA were older, more frequently female, and had a higher burden of endocrine, metabolic and gastrointestinal disorders compared to non GLP-1 RA Users; **(2)** GLP-1 RA Users showed a lower risk of all-cause death and a higher risk of acute pancreatitis, particularly in the first 6 months of therapy; **(3)** GLP-1 RA Users did not show significant differences in pancreatic cancer risk compared with Non–GLP-1 RA Users, with only a slight and time-dependent increase observed in the stratified analyses during both the early and late follow-up phases; **(4)** Subgroup analyses provided further insights into the heterogeneity of associations observed among GLP-1 RA Users, highlighting age-, sex-, and comorbidity-related differences in both mortality and the composite pancreatitis outcome.

People receiving GLP-1 RA were generally older, more often female, and had a greater prevalence of endocrine, metabolic and gastrointestinal disorders. These findings are consistent with previous real-world studies conducted among people with T2DM or obesity [13, 14].

A potential explanation lies in the therapeutic positioning of GLP-1 RA within clinical guidelines.

In T2DM, they are typically recommended as second-line or add-on therapy—most often after metformin failure—and more recently as first-line agents in people with established cardiovascular or renal disease [15, 16]. This treatment algorithm naturally selects people with longer disease duration and elevated cardiometabolic risk.

Similarly, in obesity, GLP-1 RAs—particularly liraglutide and semaglutide—are generally prescribed in cases of more severe or refractory obesity, often following failure of lifestyle interventions or other pharmacotherapies [17]. Their regulatory approval for obesity (liraglutide in 2014 and semaglutide in 2021–2022) may also contribute to the predominance of older individuals among treated people [3, 18, 19].

The higher prevalence of women among GLP-1 RA users may reflect both gender-specific healthcare behaviours and underlying differences in treatment response. Indeed, growing evidence suggests that women tend to achieve more pronounced glycaemic improvements and greater weight reduction when treated with GLP-1 RAs compared to men, partly explaining the more frequent initiation of GLP-1 therapy among female patients [20, 21].

In our study, GLP-1 RAs were associated with a significant reduction in all-cause death. This finding aligns with evidence from large trials in T2DM—such as LEADER, REWIND, and AMPLITUDE-O—which reported death reductions ranging from 12 to 22% [10, 22]. Similarly, in individuals with overweight or obesity, the SELECT trial demonstrated a 19% reduction in all-cause death with semaglutide, extending the survival benefit of GLP-1 RAs beyond diabetic populations [8, 23]. It is worth mentioning that this observation reflects class effect even though not all drugs included in the study like exenatide and lisixenatide, have proven cardiovascular benefits.

Although the direction of the association is consistent with randomized trial evidence, the more pronounced mortality reduction observed in our study should be interpreted with caution, as it may partly reflect residual confounding, differences in disease severity or concomitant therapies, and a potential healthy-user effect inherent to observational research.

Alongside these benefits, our study identified an increased risk of acute pancreatitis in GLP-1 RA Users. Though not definitively established, this association is not unexpected: prescribing information for agents like liraglutide, exenatide, and dulaglutide has long included warnings or contraindications in people with prior pancreatitis or gallstone disease [24, 25]. These precautions stem from early safety signals, including spontaneous reports, enzyme elevations, and case studies [26, 27].

Nonetheless, the causal relationship between GLP-1 RA exposure and pancreatitis remains a topic of debate. Previous meta-analyses of randomized trials have generally not demonstrated a significant increase in pancreatitis risk [28]. Recent evidence remains mixed: a meta-analysis of randomized trials reported a slight increase in pancreatitis risk among GLP-1 RA users, whereas a large real-world analysis found no excess risk in a comorbidity-free population [29, 30]. However, direct comparisons across these studies are limited by differences in design, patient selection, and follow-up duration. In contrast, our real-world cohort, without strict eligibility restrictions, may capture risk emerging in specific subpopulations.

Although a direct causal link cannot be conclusively established, several biological mechanisms support the plausibility of a connection. GLP-1 RAs are known to raise lipase and amylase levels, impair gallbladder motility, and promote bile changes—factors that may contribute to pancreatitis [27, 31]. Rodent studies have shown ductal changes and low-grade inflammation, supporting a plausible biological link [32]. These findings may also help explain why the risk of pancreatitis appears to be of greater magnitude during the first 6 months of treatment. In the LEADER trial, enzyme elevations were evident within four weeks of therapy, peaking in the early months before stabilizing (lipase + 28%, amylase + 7%) [33]. Similarly, early histologic and functional pancreatic changes were observed in animal models, suggesting an initial phase of organ stress followed by tolerance [34]. A small clinical trial of the GLP-1 RA, semaglutide reduced alcohol consumption and craving in adults with alcohol use disorder [35]. If this effect is true and consumption of alcohol is reduced through modulation of the appetite-reward centre with GLP-1 RA therapy, then the relative pancreatitis risk may be higher.

Alongside the elevated risk of acute pancreatitis, no significant differences were identified in the 1-year risk of pancreatic cancer between GLP-1 RA Users and Non–GLP-1 RA Users.

While this signal reached weak statistical significance when stratifying the follow-up into early and late phases, such findings must be interpreted with caution. Notably, similar results have been observed in previous large-scale observational studies, which also failed to establish a definitive association between GLP-1 RA use and pancreatic malignancy [28, 36].

From a pathophysiological perspective, pancreatic cancer is a disease with a prolonged latency, typically requiring years of subclinical progression before clinical manifestation [37]. It is therefore implausible that short-term GLP-1 RA exposure alone would initiate de novo malignancy within a 1-year timeframe, particularly when considering even shorter exposure periods. More likely, the malignancies identified in our study reflect a population already at higher baseline risk. Indeed, GLP-1 RA users in our cohort exhibited more frequent risk factors for pancreatic cancer—including older age, obesity, type 2 diabetes, and prior gastrointestinal disorders—suggesting a background of increased vulnerability. Moreover, a potential contribution from surveillance bias cannot be excluded. The higher incidence of pancreatitis in this group may have led to more frequent imaging and incidental detection of asymptomatic tumours (i.e., overdiagnosis and it is not uncommon for early-stage pancreatic inflammation—such as that seen in acute pancreatitis—to be radiologically misinterpreted as a neoplastic lesion, further complicating the diagnostic picture [38, 39]).

Taken together, our findings do not support a direct causal role for GLP-1 RAs in pancreatic carcinogenesis, and any potential interaction with pre-existing risk factors remains speculative. From a mechanistic standpoint, chronic low-grade pancreatic inflammation—known to contribute to tumorigenesis—might represent a plausible biological pathway in select high-risk people [32].

Indeed, younger people (< 65 years) in our study experienced a more pronounced reduction in all-cause death following GLP-1 RA therapy compared to older individuals. Although large cardiovascular outcome trials have consistently shown that GLP-1 RAs, particularly semaglutide and liraglutide, reduce all-cause death in people with type 2 diabetes [9, 10], these studies did not formally stratify death outcomes by age group, limiting direct comparisons with our findings. However, the observed age-related gradient in our analysis is biologically plausible. Younger individuals typically present with fewer comorbidities and greater physiological reserve, and—critically—they are more likely to receive GLP-1 RA therapy over a longer period. The benefit of GLP-1 RAs appears to be time-dependent: in the LEADER trial, for instance, the absolute risk reduction in all-cause death became more pronounced with longer follow-up, rising from 0.3% at 1 year to 1.4% at 3 years [40]. A similar trend was observed in REWIND, where sustained benefits emerged with extended treatment exposure, suggesting that younger people, by virtue of prolonged therapy duration, may accrue a greater cumulative benefit from GLP-1 RAs [41], potentially explaining the more marked death reduction observed in this subgroup within our study.

Just as the younger age and lower comorbidity burden likely explain the more pronounced reduction in all-cause mortality observed among younger individuals, these same factors may also account for their significantly lower risk of the composite outcome—comprising acute and chronic pancreatitis—compared with older participants, who instead exhibited an increased risk. This pattern is consistent with a healthy-user bias, whereby individuals who are younger and generally healthier tend to experience more favorable outcomes regardless of treatment exposure.

In addition to age-related differences, our subgroup analyses also revealed that female people and individuals with a higher burden of comorbidities experienced greater reductions in all-cause death following GLP-1 RA therapy. This finding in women is consistent with prior reports suggesting that GLP-1 RAs may produce more pronounced weight loss and glycemic improvements in female people, potentially due to sex-specific differences in GLP-1 receptor expression, pharmacodynamics, or hormonal interactions [21].

Similarly, the amplified survival benefit observed among people with established comorbidities—such as cardiovascular or renal disease—is likely attributable to their higher baseline risk. In this context, the absolute risk reductions afforded by GLP-1 RA therapy become more pronounced, as demonstrated in multiple cardiovascular outcome trials where the benefits were most evident among those with prior disease^4,10,41^. These observations reinforce the broader principle that the impact of preventive therapies is often greatest in high-risk populations, aligning with the risk-based treatment paradigm increasingly endorsed by contemporary clinical guidelines.

## Limitations

Several limitations should be acknowledged in interpreting the findings of this study. First, its observational design precludes definitive conclusions about causality. Although rigorous propensity score matching was applied to reduce confounding, residual confounding from unmeasured variables (e.g., lifestyle factors, socioeconomic status, over-the-counter medication use) cannot be excluded. In addition, the magnitude of the 1-year mortality reduction observed in GLP-1 RA users—larger than in randomized trials—suggests that residual confounding may persist, including confounding by indication/clinical channeling and a healthy-user effect, and that estimates may be comparator-dependent. Despite propensity-score matching and two complementary post-PSM analyses (a diabetes-severity model and an active-comparator design), platform constraints on the number of covariates and unmeasured factors mean that residual bias—including potential immortal-time/time-origin bias—cannot be entirely excluded. Second, outcome ascertainment was based on administrative coding within electronic health records, which may be subject to misclassification or underreporting, particularly for diagnoses such as chronic pancreatitis or early pancreatic cancer. Third, medication exposure was inferred from prescription or dispensing records in TriNetX and may not reflect actual adherence or persistence. Information on dose, treatment duration, titration, and discontinuation were not systematically available; therefore, residual confounding by concomitant therapies cannot be excluded. Fourth, although the study leveraged a large and diverse U.S.-based population, findings may not be generalizable to other healthcare systems or regions with different prescribing patterns and surveillance intensity. Fifth, the 1-year follow-up may not be sufficient to capture long-term pancreatic outcomes, particularly cancer, which typically has a long latency period. Finally, while time-stratified and subgroup analyses provide valuable insights, they remain exploratory and should be interpreted with caution given the potential for multiple testing, limited power in certain strata, and the inherent arbitrariness of fixed time-windows.

## Conclusion

The GLP-1 RAs in people with obesity or type 2 diabetes are associated with a significant reduction in all-cause death, alongside an increased risk of pancreatic adverse events—particularly acute pancreatitis—most pronounced during the first 6 months of treatment. These findings highlight the importance of individualized risk–benefit assessment when prescribing GLP-1 RAs, especially in people with underlying pancreatic vulnerability or elevated baseline risk.

## Supplementary Information

Below is the link to the electronic supplementary material.

Supplementary Material 1

## Data Availability

The dataset(s) supporting the conclusions of this article were obtained from the TriNetX platform (https://trinetx.com). Access to TriNetX data is available under license to participating institutions; restrictions apply to the availability of these data to non-academics. Researchers interested in accessing TriNetX data may contact TriNetX directly.
