# Supplementary material for: Risk of all–cause death and pancreatic events following GLP-1 RA initiation in people with obesity or type 2 diabetes: observations from a federated research network
Source: Cardiovasc Diabetol. 2025 Nov 19;24:438. doi: 10.1186/s12933-025-02986-0 (PMC12628983; doi:10.1186/s12933-025-02986-0)
Supplement: Supplementary file 1 — Supplementary Material 1 [file 12933_2025_2986_MOESM1_ESM.docx]

**Risk of All-Cause Death and Pancreatic Events Following GLP-1 Initiation in People with Obesity or Type 2 Diabetes: Observations from a Federated Research Network**

Enrico Tartaglia, Tommaso Bucci, Michele Rossi, Andrea Galeazzo Rigutini, Amir Askarinejad, Uazman Alam, Katarzyna Nabrdalik, Giuseppe Boriani, Gregory Y. H. Lip

Supplementary material

***Supplementary Methods***

TriNetX Database

The TriNetX data are collected from member healthcare organizations (HCO) and originates from their primary electronic health records (EHR) system. A typical HCO is a large academic health center with data coming from majority of its affiliates. A single HCO frequently has more than one facility, including main and satellite hospitals. The data are stored on the TriNetX database via a physical server at the institution’s data centre or a virtual hosted appliance. The TriNetX platform comprises of a series of these appliances connected into a federated network. This network can broadcast queries to each appliance. Results are subsequently collected and aggregated. Once the data are sent to the network, it is mapped to a standard and controlled set of clinical terminologies and undergoes a data quality assessment including ‘data cleaning’ that rejects records which do not meet the TriNetX quality standards. The TriNetX database performs internal and extensive data quality assessment with every refresh based on conformance, completeness, and plausibility (http://doi.org/10.13063/2327-9214.1244). HIPAA (Health Insurance Portability and Accountability Act) compliance of the clinical patient data is achieved using deidentification. Available data types within the network include demographics, diagnoses (represented by ICD-10-CM codes), procedures (coded in ICD-10-PCS or CPT), and measurements (coded to LOINC). While extensive information is provided about patients’ diagnoses and procedures, other variables (such as socioeconomic and lifetime factors are not comprehensively represented). The advantage of EHR data over insurance claim data is that both insured and uninsured patients are included. An advantage of EHR data over survey data is that the former represents the diagnostic rates in the population presenting to healthcare facilities. This provides an accurate account of the burden of specific diagnoses on healthcare systems. One primary limitation of relying on diagnoses is that they do not account for undiagnosed patients who might have a condition but have not yet received medical support. Another general limitation of EHR data is that a patient may be seen in different HCO for different components of their care. If one HCO is not part of the federated network, then part of their medical records may not be available. Using a network of healthcare organizations, rather than a single site, limits this possibility but does not fully remove it.

Propensity Score Matched Analyses were performed using logistic regression [Logistic Regression from the scikit-learn package in Python (version 3.7)]. TriNetX performed a 1:1 greedy nearest neighbor matching model with a caliper of 0.1 pooled standard deviations. To eliminate bias resulting from nearest neighbor algorithms, the rows were randomized. Any baseline characteristic with a standardized mean difference between cohorts lower than 0.1 was deemed well matched. (<https://www.tandfonline.com/doi/full/10.1080/00273171.2011.568786>).

Assessment of the Proportional Hazards Assumption

To evaluate whether the proportional hazards assumption was satisfied in the Cox regression models, we conducted a Chi-square (χ²) test based on Schoenfeld residuals. These tests examine whether the relationship between the associated variables and the hazard function remains stable over time. The null hypothesis posits that the effect of OAC discontinuation on the hazards of primary outcomes is constant throughout the study period. The χ² statistic measures the discrepancy between the observed and expected Schoenfeld residuals. A higher χ² value indicates a greater divergence from the expected values, suggesting a potential violation of the proportional hazards assumption. Conversely, a lower χ² value implies that the observed residuals closely align with the expected values, supporting the assumption. The p-value, derived from the χ² statistic, reflects the likelihood of observing these deviations under the null hypothesis. A p-value greater than 0.05 suggests that the deviations are likely due to random variation, indicating that the proportional hazards assumption holds. In contrast, a p-value less than 0.05 implies that the observed deviations are unlikely to be random, indicating a violation of the proportional hazards assumption.

**Supplementary Table 1**. **Inclusion and exclusion criteria in people with obesity or type 2 diabetes mellitus and a GLP-1 RA prescription compared to those without.** GLP-1 RA indicates Glucagon-Like Peptide-1 Receptor Agonist; SGLT2i indicates SGLT2 inhibitors; BMI indicates Body Mass Index; ICD-10-CM indicates International Classification of Diseases, 10th Revision, Clinical Modification; ATC indicates Anatomical Therapeutic Chemical classification system.

**Main Cohort Definitions and Characteristics**:

| Criteria | **GLP-1RA Users** | **Non–GLP-1 RA Users** | **SGLT2i Users**  *(active comparator)* |
| --- | --- | --- | --- |
| *Age* | ≥ 18 years | ≥ 18 years | ≥ 18 years |
| *Clinical Condition (Required between Jan 1, 2018 and Jan 1, 2024)* | At least one of: - BMI ≥ 30.0 kg/m² - Diabetes mellitus (ICD-10-CM: E11) | At least one of: - BMI ≥ 30.0 kg/m² - Diabetes mellitus (ICD-10-CM: E11) | At least one of: - BMI ≥ 30.0 kg/m² - Diabetes mellitus (ICD-10-CM: E11) |
| *Medication Use* | Prescription of GLP-1 RA (ATC: A10BJ) | No prescription of GLP-1 RA (ATC: A10BJ) | Prescription of SGLT2i (ATC A10BK) |
| *Exclusion Criteria* | Any history of: - Acute pancreatitis (K85) - Alcohol-induced chronic pancreatitis (K86.0) - Other chronic pancreatitis (K86.1) - Malignant neoplasm of pancreas (C25) | Same as GLP-1 RA Users | Any history of: - Acute pancreatitis (K85) - Alcohol-induced chronic pancreatitis (K86.0) - Other chronic pancreatitis (K86.1) - Malignant neoplasm of pancreas (C25) |

## **Subgroup Definitions:**

| **Subgroup Category** | **Definition (TriNetX Code)** |
| --- | --- |
| Age | ≥ 65 years vs. < 65 years |
| Sex | Female vs. Male |
| Tobacco Use | History of smoking (ICD-10-CM: Z72.0) vs. No history |
| Alcohol Use | History of alcohol use (ICD-10-CM: Z72.1) vs. No history |
| Hypertriglyceridemia | Presence (ICD-10-CM: E78.1) vs. Absence |
| Cholelithiasis | Presence (ICD-10-CM: K80.x) vs. Absence |
| Heart Failure | Presence (ICD-10-CM: I50.x) vs. Absence |
| Chronic Kidney Disease | Presence (ICD-10-CM: N18.x) vs. Absence |

**Supplementary Table 2. ICD-10-CM codes for the 1-year risk of all-cause death, the composite outcome, acute pancreatitis, chronic pancreatitis and pancreatic cancer.** ICD-10-CM indicates International Classification of Diseases, 10th Revision, Clinical Modification.

| **Outcome** | **ICD-10-CM Description** |
| --- | --- |
| *All-cause death* | Deceased (demographic status field, no ICD-10-CM code) |
| *Composite outcome* | Acute pancreatitis (K85) OR Other chronic pancreatitis (K86.1) |
| *Acute pancreatitis* | Acute pancreatitis (K85) |
| *Chronic pancreatitis* | Other chronic pancreatitis (K86.1) |
| *Pancreatic cancer* | Malignant neoplasm of pancreas (C25) |

**Supplementary Table 3.** **Risks of primary and secondary outcomes in people with obesity or type 2 diabetes mellitus and a GLP-1 RA prescription compared to those without.** GLP-1 RA indicates Glucagon-Like Peptide-1 Receptor Agonist; CI indicates Confidence Intervals; HR, Hazard Ratio. A high χ2 suggests a greater deviation from the expected values, indicating a potential violation of the proportional hazard assumption. Conversely, a small χ2 value indicates that the observed residuals closely match the expected values.

| ***Outcomes*** | ***HR (95%CI)*** | ***χ^2^ (p value)*** |
| --- | --- | --- |
| All cause death (overall) | 0.554 (0.542, 0.566) | 228.800 (< 0.001) |
| All cause death (early) | 0.484 (0.470, 0.498) | 0.784 (0.408) |
| All cause death (late) | 0.646 (0.627, 0.666) | 5.487 (0.079) |
| Composite outcome (overall) | 1.062 (1.023, 1.102) | 23.638 (< 0.001) |
| Composite outcome (early) | 1.216 (1.126, 1.313) | 4.145 (0.062) |
| Composite outcome (late) | 1.010 (0.955, 1.063) | 0.011 (0.915) |
| Acute pancreatitis (overall) | 1.058 (1.015, 1.103) | 14.956 (< 0.001) |
| Acute pancreatitis (early) | 1.143 (1.055, 1.239) | 1.386 (0.239) |
| Acute pancreatitis (late) | 1.097 (0.944, 1.162) | 0.019 (0.890) |
| Chronic pancreatitis (overall) | 0.973 (0.906, 1.045) | 14.190 (< 0.001) |
| Chronic pancreatitis (early) | 0.872 (0.795, 1.147) | 2.413 (0.120) |
| Chronic pancreatitis (late) | 0.977 (0.891, 1.072) | 0.414 (0.520) |
| Pancreatic cancer (overall) | 1.031 (0.952, 1.116) | 14.598 (< 0.001) |
| Pancreatic cancer (early) | 1.170 (1.010, 1.357) | 6.292 (0.012) |
| Pancreatic cancer (late) | 1.109 (1.006, 1.223) | 1.605 (0.072) |

**Supplementary Table 4.** **Risks of primary and secondary outcomes during the first 3 and 9 months in people with obesity or type 2 diabetes mellitus and a GLP-1 RA prescription compared to those without.** GLP-1 RA indicates Glucagon-Like Peptide-1 Receptor Agonist; CI indicates Confidence Intervals; HR, Hazard Ratio.

| ***Outcomes*** | ***HR (95%CI)*** |
| --- | --- |
| All cause death (0-3) | 0.416 (0.399, 0.433) |
| All cause death (0-9) | 0.485 (0.471, 0.500) |
| Composite outcome (0-3) | 1.062 (1.023, 1.102) |
| Composite outcome (0-9) | 1.005 (0.955, 1.058) |
| Acute pancreatitis (0-3) | 1.011 (0.898, 1.036) |
| Acute pancreatitis (0-9) | 1.012 (0.957, 1.071) |
| Chronic pancreatitis (0-3) | 0.790 (0.700, 0.891) |
| Chronic pancreatitis (0-9) | 0.859 (0.779, 0.946) |
| Pancreatic cancer (0-3) | 0.931 (0.823, 1.054) |
| Pancreatic cancer (0-9) | 0.917 (0.828, 1.015) |

**Supplementary Table 6.** **Risks of primary and secondary outcomes in people with obesity or type 2 diabetes mellitus and a GLP-1 RA prescription compared to those without.** Hazard Ratios (HR) are reported with 95% confidence intervals (CI) shown in parentheses.GLP-1 RA indicates Glucagon-Like Peptide-1 Receptor Agonist. Hazard Ratios (HR) are reported with 95% confidence intervals (CI) shown in parentheses.

|  |  | **All cause death** | |  | **Composite outcome** | |
| --- | --- | --- | --- | --- | --- | --- |
| **Subgroups** | HR (95% CI) | | *P int* | HR (95% CI) | | *P int* |
| ≥ 65 years | 0.625 (0.609, 0.641) | | 0.00 | 1.211 (1.138, 1.288) | | 0.00 |
| < 65 years | 0.434 (0.417, 0.451) | |  | 0.938 (0.893, 0.986) | |  |
| Females | 0.522 (0.506, 0.539) | | 0.00 | 1.036 (0.984, 1.091) | | 0.48 |
| Males | 0.589 (0.572, 0.606) | |  | 1.083 (1.022, 1.147) | |  |
| Smoking | 0.506 (0.475, 0.540) | | 0.00 | 1.219 (1.091, 1.360) | | 0.15 |
| No Smoking | 0.574 (0.561, 0.587) | |  | 1.055 (1.012, 1.099) | |  |
| Alcohol | 0.445 (0.399, 0.496) | | 0.00 | 0.951 (0.796, 1.135) | | 0.37 |
| No Alcohol | 0.569 (0.557, 0.582) | |  | 1.109 (1.066, 1.154) | |  |
| Triglicerides | 0.596 (0.521, 0.681) | | 0.90 | 1.158 (0.967, 1.387) | | 0.09 |
| No Triglicerides | 0.587 (0.574, 0.600) | |  | 1.105 (1.062, 1.150) | |  |
| Cholelithiasis | 0.460 (0.415, 0.510) | | 0.00 | 1.143 (0.959, 1.363) | | 0.33 |
| No cholelithiasis | 0.589 (0.576, 0.602) | |  | 1.110 (1.067, 1.155) | |  |
| Heart failure | 0.572 (0.546, 0.599) | | 0.00 | 1.026 (0.897, 1.175) | | 0.66 |
| No Heart failure | 0.614 (0.599, 0.629) | |  | 1.134 (1.089, 1.181) | |  |
| Chronic kidney disease | 0.527 (0.504, 0.550) | | 0.00 | 0.999 (0.893, 1.118) | | 0.21 |
| No chronic kidney disease | 0.614 (0.598, 0.629) | |  | 1.146 (1.099, 1.195) | |  |

**Supplementary Table 5. Risks of primary and secondary outcomes among people with obesity or type 2 diabetes mellitus who received a GLP-1 receptor agonist prescription (GLP-1 RA Users) compared with those who did not (Non–GLP-1 RA Users) or with SGLT2 inhibitor Users.** PSM indicates Propensity Score Matching; HR indicates Hazard Ratio; CI indicates Confidence Interval.

|  |  | ***Diabetes-severitymodel****  **(GLP-1 RA Users vs Non–GLP-1 RA Users)**  **After PSM** | |  |  | ***Active comparator analysis***  **(GLP-1 RA Users vs SGLT2 inhibitor Users)**  **After PSM** | |  |
| --- | --- | --- | --- | --- | --- | --- | --- | --- |
|  | GLP-1 Users (1,507,683) | Non–GLP-1 Users (1,507,683) | HR (95% CI) | | GLP-1 Users (1,495,316) | Non–GLP-1 Users (1,495,316) | HR (95% CI) | |
| All-cause death | 12654 | 35474 | 0.363 (0.355, 0.370) | | 13,072 | 18,628 | 0.694 (0.679, 0.710) | |
| Composite outcome | 5196 | 5034 | 1.047 (1.007, 1.088) | | 6805 | 6202 | 1.086 (1.049, 1.124) | |
| Acute pancreatitis | 4286 | 4072 | 1.068 (1.023, 1.114) | | 5298 | 4975 | 1.054 (1.014, 1.096) | |
| Chronic pancreatitis | 1349 | 1467 | 0.933 (0.867, 1.005) | | 2219 | 1884 | 1.166 (0.970, 1.240) | |
| Pancreatic cancer | 1163 | 1229 | 0.953 (0.880, 1.033) | | 1448 | 1462 | 0.981 (0.912, 1.055) | |

* The diabetes-severity model compares GLP-1 RA Users with Non–GLP-1 RA Users and includes adjustment for diabetes severity and diabetes-related complications (as described in the *Methods*)
